# Recent Advances in Polysaccharides from *Sophora flavescens* Ait.: Extraction, Purification, Structural Characteristics, Pharmacological Effects, Structure-Activity Relationships, and Potential Applications

**DOI:** 10.3390/molecules31193450

**Published:** 2026-09-28

**Authors:** Gang Wang, Han Di, Xinxin Chen, Yanhong Wang, Feng Guan

**Affiliations:** 1 School of Pharmacy, Heilongjiang University of Chinese Medicine, Harbin 150040, China; 15536357796@163.com (G.W.); 15846488827@163.com (H.D.); xin345187930@163.com (X.C.); 2 Key Laboratory of Basic and Application Research of Beiyao, Ministry of Education, Heilongjiang University of Chinese Medicine, Harbin 150040, China

**Keywords:** *Sophora flavescens* polysaccharides, extraction, structural characteristic, pharmacological activity, structure-activity relationship

## Abstract

Ethnopharmacological relevance: *Sophora flavescens* Ait. (*S. flavescens)* is a classical traditional Chinese medicine (TCM) with a long history of clinical use for clearing heat, drying dampness, killing parasites, and promoting diuresis. In recent years, extensive studies have confirmed that *S. flavescens* polysaccharides (SFP) are a critical bioactive macromolecular component of this plant, exerting broad and significant multi-target pharmacological effects. However, a comprehensive understanding of their structural complexity and molecular mechanisms is required to overcome current hurdles in their clinical translation. Aim of the review: This review aims to offer a systematic summary of the recent progress in the extraction, purification, detailed structural characteristics, and various pharmacological activities of SFP. It further explores the structure–activity relationships and toxicological profiles, thereby laying a solid foundation for the future development and utilization of SFP in pharmaceutical applications. Materials and methods: Key terms including “*Sophora flavescens* Ait.”, “*S. flavescens*” and “*Sophora flavescens* Ait. polysaccharide” were systematically searched in databases such as PubMed, Web of Science, and Google Scholar (2000–2026) to ensure the comprehensiveness and accuracy of the retrieved literature. Results: Modern extraction methodologies, including ultrasound-assisted, enzyme-assisted, and microwave-assisted extraction, significantly enhance SFP yield and preserve bioactivity compared to conventional hot-water extraction. Structurally, SFP are highly heterogeneous macromolecules predominantly composed of glucose, galactose, arabinose, and xylose. The diverse biological activities of SFP are heavily dependent on their structure–activity relationships; notably, targeted chemical modifications like sulfation drastically amplify their receptor affinity and efficacy. Pharmacologically, SFP exhibit potent antioxidant, anti-inflammatory, immunomodulatory, antitumor, and hepatoprotective properties by modulating core cellular signaling pathways such as NF-κB and Nrf2, while maintaining a highly favorable safety and cytocompatibility profile.

## 1. Introduction

*Sophora flavescens* Ait. is a perennial plant (Figure 1, showing the whole plant morphology) belonging to the genus *Sophora* in the family Fabaceae. Its dried roots, known as “Kushen” in TCM, have a long history of medicinal use in China and were first documented in the *Shennong Bencao Jing*. As a classical TCM, *S. flavescens* is characterized by the properties of clearing heat, drying dampness, killing parasites, and promoting diuresis [1,2,3]. It is traditionally described as bitter and cold in nature and is believed to act on the Heart, Liver, Stomach, Large Intestine, and Bladder meridians. Clinically, it is widely used to treat dysentery, hematochezia, jaundice with dysuria, leukorrhea, genital pruritus, eczema, damp sores, skin itching, scabies, and tinea infections. It is also applied externally for trichomonas vaginitis [4].

The chemical composition of *S. flavescens* is complex and diverse. Among its constituents, alkaloids, particularly matrine and oxymatrine, have long been regarded as the principal bioactive components and have been extensively investigated [5]. However, in recent years, with a deepening understanding of the multi-component, multi-target nature of TCM and the rapid advancement of natural polysaccharide research techniques, SFP, as a major active component of *S. flavescens*, has increasingly attracted significant attention from researchers [6]. Polysaccharides are widely distributed macromolecules that function not only as structural components and energy reserves but also as critical signaling molecules involved in intercellular communication, immune recognition, and biological regulation [7,8]. Compared with small-molecule alkaloids, which have well-defined structures and relatively clear targets, SFP, with their unique biological activities and complex mechanisms of action, are revealing another important dimension of *S. flavescens*’s pharmacological effects [9].

Extensive preclinical studies have demonstrated that phytochemicals of *S. flavescens* exhibit various pharmacological activities, including immunomodulation [10], antitumor effects [11], anti-inflammatory properties [12], antioxidant activity [13], liver protection, and metabolic regulation [14]. Their effects are not achieved through direct intervention at a single target but rather through systemic regulation of the body’s homeostasis via multiple pathways, such as activating macrophages, modulating lymphocyte subsets, and balancing cytokine networks [15]. Particularly in the context of antitumor activity, SFP not only directly inhibit tumor cell proliferation and induce apoptosis but also exert unique advantages by reshaping the tumor microenvironment and enhancing the body’s immune function—a mechanism that is consistent with the holistic treatment philosophy of TCM [11].

While recent studies on SFP have shown great promise, several challenges remain in advancing this field. One key obstacle is the structural complexity of polysaccharides, which includes factors such as molecular weight, monosaccharide composition, glycosidic linkages, and higher-order structures [16]. This complexity makes detailed characterization difficult, and the current methods for structural elucidation remain inadequate. Another significant issue is the incomplete understanding of how these polysaccharides exert their biological effects, especially in terms of receptor interactions and the signaling pathways involved in immune cells [17]. Moreover, translating the findings from laboratory studies into clinical applications is hindered by a lack of pharmacokinetic and safety data [18].

This review aims to provide a comprehensive overview of recent progress in SFP research. It will cover key topics such as methods for extraction, purification, and structural characterization, as well as the pharmacological activities and the molecular mechanisms behind these effects. Additionally, we will explore the structure–activity relationships of SFP, discuss existing challenges and offer insights into future directions. To illustrate these points, a schematic diagram of the extraction and purification process for SFP is provided in Figure 2. Detailed information on extraction and purification is listed in Table 1.

To ensure a comprehensive and highly reproducible evaluation, a systematic literature search was conducted across PubMed, Web of Science, CNKI, and Google Scholar spanning from January 2000 to 2026, with the final search executed on 15 July 2026. The search strategy utilized a complete set of primary keywords (“*Sophora flavescens* Ait.”, “*S. flavescens*”, and “Kushen”) systematically combined with secondary terms using Boolean operators (AND/OR): “polysaccharide”, “macromolecule”, “extraction”, “purification”, “structural characterization”, “pharmacological activity”, and “structure-activity relationship”. The inclusion criteria were strictly defined as follows: (1) original research articles and comprehensive reviews focusing on SFP; (2) studies detailing the extraction, structural characterization, or biological activities of SFP; and (3) articles published exclusively in English or Chinese. The exclusion criteria comprised: (1) studies solely focused on small-molecule alkaloids or flavonoids without addressing polysaccharides; and (2) conference abstracts, unpublished data, or articles without full-text access. During the screening process, two independent researchers initially evaluated the titles and abstracts for relevance, followed by a rigorous full-text assessment of the pre-selected papers to determine final eligibility for inclusion in this review.

## 2. Methods for Extracting SFP

### 2.1. Hot Water Extraction Methods

Hot water extraction (HWE) is the most classic and commonly used method for obtaining polysaccharides. This process essentially relies on the destruction of cell walls and membranes by high temperatures, as well as the weakening of hydrogen bonds in the polysaccharides by water molecules, thereby enabling the extraction of polysaccharides [24]. Traditional extraction methods are simple to operate and cost-effective, making them widely used for extracting polysaccharides. While numerous studies on other fungal and botanical macromolecules have systematically optimized the hot water extraction process using response surface methodology [25], Traditional hot water extraction of SFP has been extensively optimized to improve recovery efficiency. Zhang et al. isolated four water-soluble polysaccharide fractions (SF1-SF4) from *S. flavescens* roots using HWE followed by ethanol precipitation, with a total polysaccharide yield of 7.28% [6]. During the extraction process, the material is air-dried to minimize the impact of the plant’s own moisture on polysaccharide content and is thoroughly ground to enhance the release rate of polysaccharides from the cells. Another team optimized the traditional hot-water extraction process via orthogonal experiments, achieving a yield of 3.07% under the following conditions: 85 °C, a solid-to-liquid ratio of 1:35, and an extraction time of 3.5 h [19]. However, it should be noted that the traditional hot-water extraction method involves long extraction times, low yields, and high temperature requirements. To address these issues, novel and efficient extraction methods have been developed. Consequently, thermal-alkali extraction, enzyme-assisted extraction, ultrasound-assisted extraction, and microwave-assisted extraction are being increasingly applied in the extraction of SFP [26].

### 2.2. Alkali Extraction Methods

Alkali extraction of SFP is a technique that has garnered significant attention in recent years for the separation and purification of plant polysaccharides. An alkaline environment disrupts structures such as hemicellulose and lignin in plant cell walls and promotes the breaking of hydrogen bonds between polysaccharide molecules, thereby more effectively releasing water-insoluble or bound polysaccharide components [27]. Extrapolating from research on other medicinal plants (such as Panax ginseng), sodium hydroxide is the most commonly used alkaline extractant, where concentration, temperature, and duration serve as key parameters [28]. The direct application and comprehensive optimization of these generalized parameters specifically for SFP represents an ongoing area of research. Drawing upon findings from other macromolecular systems, such as mushroom polysaccharides, it is widely recognized that alkaline environments can significantly improve yields compared to traditional hot-water methods. However, these background studies also caution that excessively high alkali concentrations or prolonged extraction times may lead to the cleavage of β-glycosidic bonds, triggering degradation reactions that reduce molecular weight and biological activity [29]. This established paradigm provides critical theoretical guidance for the ongoing optimization of SFP extraction.

Compared to single-step alkali extraction, some studies have adopted a stepwise extraction strategy, namely, first performing HWE to obtain water-soluble polysaccharides, followed by alkali extraction of the residue, thereby obtaining polysaccharide fractions with different properties and activities [29]. However, polysaccharides obtained via alkaline extraction often contain higher levels of uronic acids and proteins in their chemical composition, which is related to the increased solubilization of cell wall matrix components during the extraction process; these structural features are also closely associated with their biological activities [30]. Although the alkaline extraction method can improve yield, its potential effects on polysaccharide structural modification and activity still require detailed characterization using techniques such as nuclear magnetic resonance and high-performance gel permeation chromatography [31]. Recent trends indicate that acidic and alkali extraction methods not only improve yields but also induce beneficial structural modifications, such as lower molecular weights and enriched uronic acid contents. These solvent-driven changes frequently lead to enhanced biological activities, providing a critical strategy for optimizing the structure–activity relationships of polysaccharides [32].

### 2.3. Ultrasound-Assisted Extraction Methods

As an efficient and environmentally friendly extraction technique, ultrasonic-assisted extraction demonstrates significant advantages in the extraction of SFP [33]. Its core mechanism of action primarily relies on the cavitation, mechanical, and thermal effects generated by ultrasound. These effects act synergistically on plant tissues, thereby substantially improving the extraction efficiency of target components at relatively low temperatures and within a short timeframe [34]. To maximize the yield of *S. flavescens* extract and preserve the integrity of its macromolecular structure, researchers typically use statistical methods such as response surface methodology to optimize key extraction parameters. Empirical studies have shown that extraction time (typically optimized to around 30 min), ultrasonic power (e.g., 500 W), solvent concentration, and liquid-to-solid ratio (e.g., 1:30 g/mL) are key factors that significantly influence the final yield [35,36]. Compared to traditional heating and reflux or prolonged maceration techniques, ultrasonic-assisted extraction demonstrates advantages in the preparation of polysaccharides from TCM. This method not only significantly reduces energy consumption but also enables efficient extraction at lower temperatures, thereby effectively preventing the thermal degradation of polysaccharide glycosidic bonds and better preserving the important biological activities of SFP, such as antioxidant, immunomodulatory, and anti-biofilm properties [37,38].

### 2.4. Microwave-Assisted Extraction Methods

Due to its high efficiency, high speed, and energy-saving characteristics, microwave-assisted extraction has become a modern method for enhancing the extraction efficiency of SFP. This technology utilizes microwave energy to directly act on polar molecules within the material, generating instantaneous heat that creates pressure inside the cells, thereby rapidly disrupting the cellular structure and promoting the elution of active components such as polysaccharides [39]. Zhang et al. used MAE with water as the extraction solvent for *S. flavescens* roots, optimizing parameters such as microwave power, extraction time, and temperature. They found that extraction at 400 W for 20 min at 85 °C with a liquid-to-solid ratio of 30:1 achieved a polysaccharide yield of 11.6%, demonstrating that MAE can substantially improve extraction efficiency compared with traditional hot-water methods [6]. Although traditional hot-water extraction is a mild method, it is typically time-consuming and yields limited results. Microwave-assisted extraction is not only more efficient, but its combination with the two-phase system also utilizes the phase separation and polarity differences in the ethanol/ammonium sulfate system to simultaneously achieve extraction and preliminary separation and purification, thereby simplifying subsequent processes [40]. However, during microwave treatment, precise control of time and power is essential to avoid polysaccharide degradation caused by localized overheating, thereby ensuring the preservation of their structure and biological activity [41].

### 2.5. Enzyme-Assisted Extraction Methods

Enzyme-assisted extraction (EAE) is an efficient, eco-friendly, and mild technique increasingly used for polysaccharide recovery. By utilizing specific degrading enzymes, such as cellulase and pectinase, EAE effectively breaks down the complex plant cell wall matrix of *Sophora flavescens*. This enzymatic hydrolysis reduces mass transfer resistance, facilitating the release of intracellular compounds and significantly enhancing the polysaccharide yield [42]. EAE is typically performed under mild conditions (e.g., 40–50 °C, slightly acidic pH) to maintain optimal enzyme activity and prevent the cleavage of glycosidic bonds that can occur during conventional HWE [43]. Given its ability to significantly reduce extraction time and energy consumption while better preserving the molecular weight, spatial conformation, and biological activities such as antioxidant properties, developing a targeted enzyme-assisted extraction method for SFP represents a highly promising and necessary approach [44].

### 2.6. Comparative Analysis of Extraction Efficiencies

To transition from a descriptive overview to a critical evaluation, it is imperative to compare these methodologies specifically in the context of SFP regarding yield, bioactivity retention, and economic efficiency. Conventional HWE is the most economically burdensome method, requiring substantial energy inputs (e.g., continuous heating at 85–100 °C for up to 3.5 h) while delivering suboptimal yields ranging from 2.50% to 4.83% [19,21]. Furthermore, the prolonged thermal exposure inherent to HWE poses a critical risk of degrading the sensitive glycosidic bonds in SFP, which can diminish the macromolecule’s native pharmacological activities. In contrast, modern extraction techniques significantly optimize this cost–benefit ratio. MAE demonstrates high economic efficiency in terms of time, achieving the highest reported SFP yield (11.6%) in merely 20 min. However, MAE requires precise parameter control to mitigate the risk of localized thermal degradation. When evaluating the optimal balance of structural preservation and extraction efficiency for SFP, mechanochemistry-assisted extraction exhibits profound advantages. By employing mechanical milling at room temperature for just 10 min, this technique bypasses thermal stress entirely, thereby maximizing the retention of the polysaccharides’ native spatial conformations and intrinsic bioactivities (e.g., antioxidant capacity). Concurrently, it achieves a highly competitive yield of 7.28%, representing a highly efficient, solvent-minimized, and economically viable strategy for SFP preparation [6]. However, despite these methodological advancements, a major overarching limitation in current extraction and purification practices is the lack of standardized protocols across laboratories, leading to pronounced batch-to-batch sample heterogeneity and severely compromising cross-study comparability.

## 3. Methods for the Isolation and Purification of SFP

### 3.1. Purification Methods

#### 3.1.1. Deproteinization Methods

Deproteinization and purification of SFP polysaccharides are critical steps for ensuring their structural integrity and for studying their biological activity. Currently, several methods are available, including the Sevag method and the TCA method.

The Sevag method is a classic deproteinization technique first proposed by Sevag and is widely used for the purification of biomacromolecules such as polysaccharides and nucleic acids. This method is relatively mild and does not easily cause degradation of the target molecule, making it particularly suitable for substances sensitive to heat or pH. While the Sevag method has proven highly effective for purifying macromolecules from other botanical sources, for instance, yielding protein-free polysaccharide fractions from Anredera cordifolia [45], detailed literature characterizing its specific efficiency and optimization exclusively for SFP remains limited. However, although the Sevag method is not the most efficient, requiring multiple repetitions to remove most proteins and resulting in some sample loss, it remains a standard deproteinization technique in laboratory preparations due to its mild conditions, simple equipment, and avoidance of harsh chemical reagents. It is often combined with other methods to obtain higher purity biomacromolecules [46].

The trichloroacetic acid (TCA) precipitation method is a highly efficient and rapid protein removal technique based on strong acid denaturation. It is widely used in the fields of biochemistry and molecular biology, particularly in nucleic acid purification, metabolomics sample preparation, and the removal of proteins from supernatants in proteomics [47]. Compared to milder methods such as the Sevag method, the concentration and treatment time must be optimized in specific applications based on the sample type and the stability of the target molecule. When extracting small RNA molecules or performing metabolite analysis, TCA is often rapidly added under low-temperature conditions followed by immediate centrifugation to minimize degradation of the target compounds [48].

#### 3.1.2. Decolorization Methods

Decolorization is one of the key steps in polysaccharide purification. Therefore, selecting a decolorization method that is efficient, gentle, and maximizes the preservation of polysaccharide structure and activity is crucial. Currently, decolorization techniques applied to polysaccharide systems are primarily based on adsorption and oxidation methods [49]. These generalized background principles are actively being adapted for SFP purification. The adsorption method is one of the most widely used polysaccharide decolorization methods; activated carbon physically adsorbs pigment molecules by leveraging its enormous specific surface area and abundant pore structure [50]. Due to its non-selective adsorption, the removal of pigments inevitably results in the loss of some polysaccharides, thereby affecting the final yield [51]. Oxidative decolorization, primarily involving hydrogen peroxide and its activation systems, achieves decolorization by destroying the chromophores of pigment molecules through strong oxidizing power. This method offers rapid decolorization and simple post-treatment. However, its harsh oxidative conditions trigger the oxidative degradation of polysaccharide chains, leading to a decrease in their biological activity, which in turn reduces the decolorization efficiency [52].

#### 3.1.3. Removing Other Impurities Methods

In the purification process of SFP, the ethanol precipitation method has long been regarded as the core step for the preliminary separation and enrichment of polysaccharides from crude extracts due to its clear mechanism, simple operation, and ease of scaling up [53]. This method primarily relies on the significantly reduced solubility of polysaccharides in an ethanol-water mixed solvent. By altering the solution’s polarity and dielectric constant, it promotes the dehydration and aggregation of polysaccharide molecules, causing them to precipitate and separate from most water-soluble small-molecule impurities [54].

### 3.2. Separation Methods

The recovery of crude SFP primarily relies on the ethanol precipitation method [54]. Studies have shown that adding four times the volume of anhydrous ethanol to a concentrated water extract of *S. flavescens*, followed by overnight static incubation at low temperature and centrifugation, can effectively recover the polysaccharide precipitate [23]. After obtaining the crude polysaccharide, further purification is required to obtain a homogeneous fraction. Among column chromatography techniques, ion-exchange chromatography and gel filtration chromatography are the most widely used. Ion-exchange chromatography primarily relies on differences in the charges carried by polysaccharides for separation [55].

SFP ion-exchange chromatography using a DEAE-52 cellulose column is commonly employed to fractionate the crude polysaccharides [51]. Stepwise elution with NaCl solutions of increasing concentration allows the sequential separation of neutral and acidic polysaccharide fractions, with the acidic fractions typically containing uronic acid residues. To further purify these fractions and obtain polysaccharides with relatively uniform molecular weights, gel filtration chromatography (e.g., Sephadex G-100 or G-200) is frequently used. This method separates molecules based on differences in their hydrodynamic volume, allowing for the isolation of homogeneous polysaccharide fractions suitable for structural characterization and bioactivity assays [56].

## 4. Structural Characterization

Structural complexity of SFP is determined by their diverse molecular weights, monosaccharide compositions, and intricate glycosidic linkage patterns, which are significantly influenced by extraction and purification protocols [57].

### 4.1. Molecular Weight

The molecular weight distribution of SFP polysaccharides is one of the key parameters in their structural characterization. It not only directly influences the physicochemical properties of the polysaccharides, such as solubility and viscosity, but is also closely related to their biological activity [15]. Studies have shown that crude SFP fractions are predominantly high-molecular-weight heteropolysaccharides, with molecular weights typically ranging from tens of thousands to millions of daltons, exhibiting a certain degree of polydispersity. This broad distribution range primarily stems from the inherent heterogeneity of the plant source, differences in the extracted parts, and variations in extraction and purification processes [52].

Early studies often employed gel filtration chromatography (GFC) combined with standard reference comparison to estimate molecular weight. While this method provides relative molecular weight information, its accuracy is significantly affected by differences in the structure of the standards and the conformation of the polysaccharides under analysis. In recent years, high-performance gel permeation chromatography (HPGPC) coupled with a multi-detector system has become the primary method for determining the absolute molecular weight of SFP. Among these, the combination of HPGPC with a multi-angle laser light scattering (MALLS) detector and a refractive index (RI) detector is particularly crucial. The MALLS detector does not rely on reference standards and can directly measure information such as the weight-average molecular weight and root-mean-square radius of gyration of macromolecules in solution, thereby determining the absolute molecular weight. Studies have shown that the weight-average molecular weights of different SFP fractions measured by this method vary significantly. The molecular weight of neutral polysaccharide fractions purified from *S. flavescens* roots is approximately 120 kDa, while that of certain acidic polysaccharide fractions can reach 500 kDa. Specific studies have also isolated homogeneous fractions such as SP-1-1/2 (85 kDa) SFWP (51 kDa) [23] and various sub-fractions from water decoctions ranging from 3.4 to 150 kDa [20].

Additionally, the homogeneity and molecular weight (MW) of SFP significantly impact their bioactivities. SFP fractions with higher MW and larger spatial structures exhibit greater potential in stimulating macrophage phagocytosis and upregulating TNF-*α* secretion in vitro at doses of 50–250 mg/L. Specifically, fractions with a peak MW exceeding 100 kDa (e.g., a highly active 102 kDa fraction) demonstrate prominent, dose-dependent promotion of T- and B-lymphocyte proliferation. However, MW is not the sole determinant; it synergizes with specific monosaccharide compositions—such as an Ara:Glc:Rha:Gal molar ratio of 1.00:0.06:0.02:0.29 found in potent fractions—and glycosidic configurations to constitute the structural basis for immunological efficacy [20].

### 4.2. Monosaccharide Composition

The monosaccharide composition of purified SFP fractions forms the basis of their structural characterization, directly determining the primary structural framework, physicochemical properties, and biological activity of the polysaccharides. Studies have shown that SFP are not single-component substances but rather heteropolysaccharides composed of various monosaccharides in specific proportions; the types and molar ratios of these components exhibit certain variations depending on the plant’s origin, the part from which it is extracted, and the purification process. Li’s team determined the monosaccharide composition of the purified polysaccharides in the form of alcohol acetate esters using GC–MS. SFNP-1 and SFNP-2 are primarily composed of glucose, with small amounts of arabinose (Ara) and galactose (Gal), as well as trace amounts of xylose. Overall, the monosaccharide composition primarily consists of neutral sugars, uronic acids, and trace amounts of amino sugars. Neutral sugars are the core components, commonly including glucose, galactose, arabinose, rhamnose, mannose, xylose, and fucose. Among these, glucose and galactose have been reported as the major components in many studies, while arabinose and rhamnose are also widely present. A major water-soluble polysaccharide isolated from *S. flavescens* roots by Chen’s team was analyzed to contain glucose, galactose, arabinose, and rhamnose, with a molar ratio of approximately 3.5:2.1:1.0:0.8. SFP are primarily heteropolysaccharides composed of neutral sugars and uronic acids. Analysis via GC-MS and HPLC indicates that glucose (Glc), galactose (Gal), and arabinose (Ara) are the dominant neutral monosaccharides, followed by rhamnose (Rha), mannose (Man), and xylose (Xyl) [6,20,23]. The presence of galacturonic acid (GalA) and glucuronic acid (GlcA) is a hallmark of the acidic fractions (SFAP), where GalA serves as the core component of the pectic backbone.

### 4.3. Structural Characteristics

The polysaccharides isolated from *S. flavescens* exhibit highly diverse and distinct structural characteristics depending on the extraction and purification methods. For instance, the four fractions (SFNP-1, SFNP-2, SFAP-1, and SFAP-2) isolated by Li et al. present a clear dichotomy between neutral and acidic forms. Their primary structural feature is a homogalacturonic acid backbone composed of *α*-(1→4)-linked galacturonic acid (GalA*p*), with approximately half of the galacturonic acid residues being methylated, with methylation degrees of 57.4% and 55.2%, respectively. SFNP-1 and SFNP-2 are neutral polysaccharides primarily composed of a backbone of (1→4)-linked glucose residues, with a small amount of side-chain structures branching at the O-4 position. This glucan-dominant framework aligns with the findings of Olennikov et al., who characterized SP-1-1/2 as an 85 kDa-(1→4)-glucan partially substituted at the C-6 position by single glucopyranose units.

On the other hand, SFAP-1 and SFAP-2 are acidic polysaccharides belonging to the pectin-type family. Their primary structural feature is a homogalacturonic acid (HG) backbone composed of *α*-(1→4)-linked galacturonic acid (GalA*p*), with approximately half of the galacturonic acid residues being methylated (methylation degrees of 57.4% and 55.2%, respectively) [23]. The high heterogeneity of these acidic fractions is further corroborated by Chen et al., who isolated multiple sub-fractions (e.g., SFP-100-B-a) with peak molecular weights reaching 102 kDa and specific molar ratios of arabinose, glucose, rhamnose, and galactose. Moreover, Zhang et al. characterized four acidic heteropolysaccharides (SF1-SF4) using IR spectroscopy, not only confirming the coexistence of both—and *β*-glycosidic bonds but also indicating that their sugar rings predominantly exist in the furanose form.

Through a comprehensive analysis involving methylation studies, partial acid hydrolysis, and NMR spectroscopy, particularly 1D and 2D NMR techniques such as ^1^H-NMR, ^13^C-NMR, COSY, HSQC, and HMBC, to elucidate the linkage patterns of their glycosidic bonds. Common backbone structures include 1,4-linked *α*-D-galacturonic acid regions (homogalacturans, HG) and highly branched RG-I regions. The main chain of the RG-I region consists of repeating [→2)-*α*-L-rhamnose-(1→4)-*α*-D-galacturonic acid-(1→] disaccharide units, often with side chains attached to the O-4 or O-3 positions of the rhamnose residue. As demonstrated by Zhao et al. sulfation modifications (yielding SHCS, SFHS, and SFAS) primarily target the C-6 hydroxyl position of the (1→)-linked main chains. This modification significantly shifts the spatial charge and alters the polysaccharide’s conformation, laying a structurally modified foundation for enhanced biological activities [20].

### 4.4. Types of Glycosidic Bonds

To date, only a few research groups have investigated the glycosidic bonds of SFP; the types of these bonds exhibit diversity and complexity across different extraction methods and purified fractions [15]. According to the research by Zhang Qihong et al., the four polysaccharide fractions (SF1, SF2, SF3, and SF4) isolated from *S. flavescens* are all acidic heteropolysaccharides composed of rhamnose, arabinose, xylose, mannose, glucose, and galactose. Infrared spectroscopy analysis indicates that these polysaccharides contain both *α*- and *β*-glycosidic bonds. Among them, SF1, SF2, and SF3 exhibit characteristic absorption peaks at 855.94 cm^−1^, 878.10 cm^−1^, and 830.61 cm^−1^, indicating the presence of *α*-glycosidic bonds, while absorption peaks at 763.50 cm^−1^, 764.12 cm^−1^, and 767.28 cm^−1^ suggest the presence of *β*-glycosidic bonds, while SF4 exhibits a characteristic absorption peak at 874.93 cm^−1^, suggesting that it primarily contains *β*-type glycosidic bonds. Furthermore, all polysaccharide components exhibit strong absorption peaks in the range of 1023.45 cm^−1^ to 1074.41 cm^−1^, indicating that their sugar ring structures exist predominantly in the furanose form [6]. In a study by Zhao Xiao liang et al., three polysaccharides (SFC, SFH, and SFA) extracted from *S. flavescens* were subjected to sulfation modification. NMR carbon spectroscopy analysis revealed that the C-6 signal of SFP shifted from δ 60.40 to δ 66.37, indicating that sulfation primarily occurred at the hydroxyl group at the C-6 position, which confirms that the C-6 position in the glycosidic bond is the primary modification site [19]. It also indicates that the glycosidic bonds in the main chains of these polysaccharides are predominantly (1→)-linked, and that the C-6 position exhibits high chemical reactivity. These distinct structural specificities lay the precise molecular foundation for their biological activity. For instance, an independent and highly cited study by Bai et al. successfully isolated a novel SFP fraction and suggested that its unique structural conformation is closely associated with its potent in vivo antitumor and immunomodulating activities [21]. As emphasized by Zeng et al. in their comprehensive review on TCM polysaccharides, this extreme diversity in glycosidic bond types, varying molecular weights, and specific monosaccharide compositions is essentially what dictates the spatial networks (e.g., furanose/pyranose rings and *α*/*β*-configurations) required to successfully recognize and bind to immune receptors [31]. The team analyzed the glycosidic linkage composition of two neutral polysaccharides using methylation analysis and GC-MS. Glcp residues linked via (1→4) bonds predominated in the glycosidic residues of SFNP-1 and SFNP-2, indicating the presence of (1→4)-linked glucans. A small number of terminal (t-) and (1→4, 6)-linked Glcp residues were observed, suggesting minor branching at the O-4 position of the main-chain residues. In addition, small amounts of t- and (1→5)-linked Araf, as well as t-, (1→3)-, (1→4)-, and (1→3, 6)-linked Galp residues were detected; these glycosidic residues may reflect the presence of impurities. A schematic diagram of its structural framework is shown in Figure 3.

### 4.5. Macromolecular Conformation and Degree of Branching

While primary structural features such as monosaccharide composition and glycosidic linkages are well-documented, the quantitative degree of branching and the three-dimensional macromolecular conformation are decisive factors for elucidating how SFP interact with pattern recognition receptors on immune cells [15]. Structural elucidation via methylation analysis reveals that neutral SFP fractions, such as SFNP-1 and SFNP-2, are primarily characterized by (1→4)-linked glucose backbones with a relatively low degree of branching, exhibiting sparse branching primarily at the O-4 position. In contrast, the acidic pectin-like fractions (e.g., SFAP-1 and SFAP-2) present highly branched rhamnogalacturonan I (RG-I) domains, contributing to a more sterically complex network [23]. Furthermore, the spatial conformation of these macromolecules in aqueous solutions strongly influences their bioaccessibility and receptor binding affinity [31]. Advanced structural analyses indicate that the major SFP fractions—particularly the neutral (1→4)-linked glucans—predominantly adopt a flexible random coil conformation rather than a rigid triple-helix structure. This dynamic random coil topology, synergizing with a low degree of branching, minimizes structural steric hindrance. Consequently, it allows the polysaccharide chains to maintain an extended conformation, maximizing the spatial exposure of key active motifs for highly specific receptor recognition and binding, such as during macrophage activation. Despite these analytical developments, structural characterization remains a critical bottleneck, as current methods are often insufficient at the level of fine architecture and higher-order conformation, which continues to restrict a comprehensive understanding of structure–activity relationships.

## 5. Pharmacological Effects

### 5.1. Antioxidant Effects

The antioxidant effect of SFP has been a focus of pharmacological research in recent years. In vitro studies have shown that both crude SFP and its sulfated derivatives possess the ability to directly scavenge various free radicals. Preliminary in vitro evaluations suggest that natural SFP fractions, typically extracted via hot water or mechanochemical-promoted technologies, exhibit concentration-dependent antioxidant activity against 2,2-diphenyl-1-picrylhydrazyl (DPPH) radicals, hydroxyl radicals, and superoxide anions [22]. However, the bioactivity of natural SFP is often constrained by its structural complexity and limited water solubility. To overcome these limitations, chemical modifications, particularly sulfation, have been widely employed to augment the structural and functional profile of natural polysaccharides [58]. The intrinsic antioxidant capacity of SFP is intimately associated with its specific monosaccharide composition—predominantly comprising glucose, mannose, galactose, and arabinose—as well as its molecular weight and complex spatial conformations [21]. The research team found that the sulfated derivative SFHS, at a concentration of 5 mg/mL, achieved scavenging rates of 69.8%, 50.4%, and 58.5% for DPPH, hydroxyl, and superoxide anion radicals, respectively, all of which exceeded those of the parent polysaccharide. The maximum DPPH radical scavenging rates for SFC and SFCS were 61.8% ± 1.1% and 63.9% ± 4.2%, respectively; the DPPH radical scavenging activities of SFH and SFHS were 66.8% ± 3.6% and 69.8% ± 6.1%; while those of SFA and SFAS were 60.6% ± 1.0% and 63.6% ± 6.3%. These findings indicate that SFC, SFH, SFA, and their sulfate derivatives possess strong DPPH radical scavenging activity, with the sulfate group enhancing this activity [59]. This activation of the intracellular endogenous antioxidant network aligns with the mechanism by which polysaccharides generally exert their effects through the regulation of endogenous antioxidant stress pathways such as Nrf2/ARE [60,61].

Moreover, sulfated modification introduces negatively charged sulfate ester groups (typically at the C-6 hydroxyl position of the sugar ring), which enhances the molecule’s hydrophilicity, expands its spatial chain conformation, and significantly improves its capacity to donate hydrogen atoms to neutralize free radicals [23]. Comparative analyses indicate that highly substituted sulfated SFP derivatives demonstrate vastly superior antioxidant efficacy compared to their unmodified counterparts, highlighting the critical role of the degree of substitution (DS) in maximizing bioactivity [20]. Consequently, the targeted structural modification of SFP presents a highly viable strategy for developing potent, natural antioxidant agents for structural characterization and future therapeutic applications. Therefore, the antioxidant mechanisms of SFP are twofold: on the one hand, they act as free radical scavengers to directly neutralize reactive oxygen species; on the other hand, they function as cellular signaling modulators to enhance the body’s own antioxidant enzyme defense system [62]. Based on their clear efficacy in improving oxidative stress, studies suggest that SFP are one of the active compounds in *S. flavescens* that combat diseases closely associated with oxidative damage, such as diabetic complications [63]. A schematic diagram of its antioxidant mechanism is shown in Figure 4.

### 5.2. Anti-Inflammatory and Analgesic Effects

As illustrated in the mechanistic overview, the downregulation of the NF-κB signaling pathway serves as a critical bridge linking the antioxidant and anti-inflammatory effects of SFP. Oxidative stress and inflammation operate through an interdependent feedback loop, where excessive reactive oxygen species (ROS) activate upstream kinases that trigger NF-κB nuclear translocation, subsequently upregulating pro-inflammatory mediators that amplify cellular oxidative stress. By suppressing NF-κB, SFP simultaneously mitigates ROS-induced oxidative damage and interrupts the inflammatory cascade, demonstrating the pleiotropic, multi-target nature typical of botanical macromolecules rather than isolated mechanisms.

Inflammation is a biological process that occurs when the body is subjected to external or internal attacks. As such, inflammation serves as a protective mechanism, shielding the body from dangerous stimuli such as infections, injuries, and oxidative stress [64]. Purified SFP fractions have demonstrated significant anti-inflammatory and analgesic activity in both in vitro and in vivo models, with their mechanisms of action primarily involving the regulation of macrophage function and the modulation of key inflammatory signaling pathways [65]. At the cellular level, these purified fractions effectively inhibit macrophage inflammatory responses induced by stimuli such as lipopolysaccharide (LPS). Specifically, they significantly reduce the production and release of pro-inflammatory mediators such as nitric oxide (NO), tumor necrosis factor-*α* (TNF-*α*), interleukin-6 (IL-6), and monocyte chemotactic protein-1 (MCP-1). The molecular mechanism underlying its anti-inflammatory action is crucial. Studies indicate that SFP exert their inhibitory effects by modulating the core signaling pathway involving NF-κB/MAPKs/c-Jun. This pathway serves as the central regulatory network of the inflammatory response; by inhibiting it, SFP reduce the transcription and expression of downstream pro-inflammatory cytokines and enzymes [23]. Furthermore, studies suggest that its anti-inflammatory effects are associated with the regulation of macrophage polarization, specifically by inhibiting pro-inflammatory M1 polarization and promoting anti-inflammatory M2 polarization, thereby reshaping the inflammatory microenvironment [66].

One specific in vivo murine study reported that a water-soluble polysaccharide (SFP, molecular weight approximately 51 kDa) purified from *S. flavescens* reduced the number of writhing episodes in an acetic acid-induced writhing model in a dose-dependent manner (100–400 mg/kg), with an inhibition rate of 42–72%, an effect comparable to that of morphine (2.5 mg/kg) [23]. In the formalin test, it significantly inhibited the pain response during the second phase (inflammatory phase, 15–30 min) but had no effect on the first phase (neurogenic phase, 0–5 min). However, another structural biology study indicated that the major acidic polysaccharides isolated from *S. flavescens* (pectin-like polysaccharides SFAP-1 and SFAP-2) did not exhibit typical anti-inflammatory effects by inhibiting NO release in an LPS-induced macrophage inflammation model; in fact, they even exhibited stimulatory effects at higher concentrations. A schematic diagram of its anti-inflammatory mechanism is shown in Figure 5.

### 5.3. Antitumor Effects

Traditionally, the dried roots of *S. flavescens* have been extensively utilized in clinical practice for the treatment of various solid tumors and inflammatory diseases. While quinolizidine alkaloids and prenylated flavonoids have historically been focalized as the primary cytotoxic agents, recent research has increasingly illuminated the indispensable role of crude SFP fractions in the herb’s holistic antitumor efficacy [67]. These polysaccharides act through a multifaceted approach, encompassing direct cytotoxicity against malignant cells, induction of apoptosis, and robust immunomodulatory enhancement. Preliminary in vitro assays suggest that these polysaccharide extracts can inhibit the proliferation of hepatocellular carcinoma (HepG2) and cervical cancer (HeLa) cells, potentially inducing targeted cellular apoptosis without inducing severe toxicity in normal cells [21]. Beyond direct cytotoxicity, the macroscopic antitumor efficacy of SFP relies heavily on the potentiation of the host’s immune defense mechanisms. These macromolecules are potent activators of macrophages, significantly upregulating phagocytic activity and stimulating the robust synthesis and release of nitric oxide. They also promote splenocyte proliferation, functioning as an immunopotentiating agent that bridges innate and adaptive immune responses against malignant microenvironments [21]. This immunomodulatory synergy provides a plausible experimental rationale for the effects observed in composite formulations in preclinical models, wherein macromolecular sugars operate in tandem with alkaloids to remodel the tumor immune microenvironment [68,69].

### 5.4. Immunomodulatory Effects

Immunomodulation represents one of the core pathways through which natural macromolecular polysaccharides exert their systemic pharmacological benefits [70]. Recent evidence underscores both crude and purified SFP fractions as potent broad-spectrum immunopotentiators that orchestrate the host immune system through multi-target and multi-pathway synergies. At the level of innate immunity, purified SFP macromolecules exhibit a profound stimulatory effect on macrophages. In vitro investigations have demonstrated that purified water-soluble fractions from *S. flavescens* roots specifically activate macrophage cell lines (e.g., RAW 264.7), significantly enhancing phagocytic capacity and inducing the expression of inducible nitric oxide synthase (iNOS). This activation triggers the robust secretion of pivotal immune effectors, including nitric oxide (NO) and tumor necrosis factor-alpha (TNF-*α*), which are fundamental for anti-inflammatory responses and the remodeling of the tumor immune microenvironment. Furthermore, SFP maintain exceptional immune-restorative capabilities in models of drug-induced immunosuppression. For instance, an *α*-glucan core component (SP-1-1/2, ~85 kDa) isolated from *S. flavescens* has demonstrated potent in vivo immunostimulating activity by effectively reversing the systemic suppression induced by agents such as azathioprine [71]. This immune-centric mechanism not only clarifies the efficacy of SFP in mitigating Concanavalin A (ConA)-induced hepatic injury and inhibiting viral replication (e.g., HBV) but also aligns with the contemporary academic consensus on utilizing plant-derived macromolecules to restore systemic immune homeostasis [10,72,73].

### 5.5. Hepatoprotective and Anti-HBV Effects

SFP demonstrate significant pharmacological value in maintaining hepatic functional homeostasis and combatting viral hepatitis, serving as a core material basis for the herb’s clinical hepatoprotective efficacy. Yang et al. reported that crude SFP provides protective effects in specific experimental murine liver injury models. For instance, in a single ConA-induced immunological liver injury model, crude SFP was observed to downregulate serum alanine aminotransferase (ALT) and aspartate aminotransferase (AST) levels and attenuates hepatic necrosis by modulating host T lymphocyte activation and suppressing the overexpression of pro-inflammatory cytokines (such as TNF-*α*, IFN-γ, and IL-6) [74]. Recent molecular pharmacological evidence further reveals that active components in *S. flavescens* can effectively block programmed cell death under stress by inhibiting the DUSP2-mediated mitochondrial apoptosis pathway, thereby achieving cellular protection at the molecular level [75].

In the antiviral domain, the inhibitory effect of SFP on the hepatitis B virus (HBV) is particularly prominent. Validated via the HepG2.2.15 cell in vitro model, SFP and its specific purified fractions can dose-dependently inhibit the secretion of HBsAg and HBeAg, and significantly downregulate HBV DNA replication levels [74,76]. This antiviral effect holds significant academic importance in the early intervention of the “hepatitis-cirrhosis-hepatocellular carcinoma” pathological deterioration axis. Notably, in herb pair studies involving combinations with *Astragalus mongholicus*, SFP demonstrates synergistic potential in remodeling the tumor immune microenvironment. Furthermore, in the widely used clinical preparation Compound Kushen Injection, the quality fingerprinting of macromolecular sugar components confirms the stability and indispensability of polysaccharide fractions in contributing to the formulation’s overall efficacy [77].

Compared to certain flavonoid components in *S. flavescens* that possess potential hepatotoxicity, macromolecular polysaccharides exhibit exceptionally high cytocompatibility and safety. This “low-toxicity, high-activity” characteristic positions them as ideal natural hepatoprotective candidate molecules [78]. Through targeted chemical modifications such as sulfation, the polyanionic properties of SFP are enhanced, substantially increasing their affinity for immune receptors like TLR4. This modification further augments their capacity to scavenge superoxide anions and protect hepatocyte membranes against lipid peroxidation [19]. This multi-dimensional protective mechanism—centered on immunomodulation and combined with direct antiviral and antioxidant activities—constitutes a promising preclinical foundation for exploring SFP as a potential candidate in chronic liver disease management [10].

### 5.6. Other Effects

Crude SFP fractions exhibit lipid-lowering effects in vitro. Existing research, primarily based on in vitro experimental models, has revealed their direct mechanisms of action. Studies have shown that crude SFP can effectively bind to bile acids in vitro. For example, at a concentration of 10 mg/mL, SFP achieved binding rates of 34.4%, 45.4%, 19.7%, and 37.7% for sodium cholate (SC), sodium taurocholate (STC), sodium glycocholate (SGC), and mixed bile salts (MBS), respectively. This binding action is similar to that of bile acid sequestrants, which reduce the intestinal reabsorption of bile acids, thereby promoting the liver’s use of cholesterol to synthesize new bile acids and indirectly lowering cholesterol levels in the body [19]. Notably, this mechanism of lipid metabolism regulation via intestinal bile acid sequestration is a classical and widespread pathway among bioactive natural macromolecules. Extensive parallel studies corroborate the universality of this mechanism. For instance, purified Stigma maydis polysaccharides (SMP-1) also display potent in vitro bile acid binding capabilities, which correlate with significant reductions in total cholesterol and triglyceride levels in hyperlipidemic animal models [79]. Furthermore, sulfated polysaccharides derived from marine brown algae have been confirmed to efficiently sequester bile acids, resulting in a marked decrease in total intracellular cholesterol in HepG2 cells in vitro, alongside excellent biocompatibility [80]. These high-quality findings in related fields provide robust mechanistic corroboration and theoretical substantiation for SFP as a promising natural candidate for hypolipidemic intervention.

Furthermore, one in vitro study reported that crude SFP exerts a dose-dependent impact on rat dental pulp stem cells (DPSCs); low doses of SFRP promote cell migration, osteogenic differentiation, and the up-regulation of osteogenic genes, whereas higher concentrations suppress DPSC proliferation, migration, and osteogenic differentiation [81]. Table 2 summarizes the activity of SFP and its potential mechanisms, and its main pharmacological activities are shown in Figure 6.

## 6. Pharmacokinetics

The aforementioned pharmacological activities are well-supported by in vitro models detailing specific molecular pathways; however, a critical bottleneck in the clinical translation of SFP is the underexplored realm of their pharmacokinetic profiles and gastrointestinal metabolism [18]. Given their nature as high-molecular-weight hydrophilic macromolecules, intact SFP are generally presumed to have poor oral bioavailability, making it difficult for them to cross the intestinal epithelium into the systemic circulation. Nevertheless, this microbiota-driven efficacy is currently speculative. It represents an extrapolation from general botanical polysaccharide research, as direct in vivo pharmacokinetic data and SFP-microbiome interaction studies are still lacking.

Recent advances in related botanical macromolecule research suggest that the systemic bioactivity of such polysaccharides may rely heavily on their indirect interactions with the gut microbiota [72]. It is postulated that because the human upper GI tract lacks the specific carbohydrate-active enzymes required to cleave complex glycosidic bonds, a significant portion of these macromolecules might reach the colon largely intact. In the colon, they could potentially serve as fermentable prebiotics for the gut microbiota. Through microbial fermentation, it is highly plausible that SFP are metabolized into absorbable bioactive metabolites, such as short-chain fatty acids (SCFAs). These SCFAs might subsequently enter the systemic circulation to exert profound anti-inflammatory and immunomodulatory effects. Therefore, the macroscopic pharmacological benefits of SFP observed in vivo are likely not solely mediated by direct receptor binding of intact polysaccharides, but also synergistically driven by the host-microbial metabolic axis. Future research must prioritize in vivo pharmacokinetic tracking and microbiome and metabolomic profiling to fully validate this indirect biological pathway. Furthermore, the severe paucity of systematic pharmacokinetic data and in vivo tracking remains a major barrier, making it difficult to bridge preliminary cellular or animal findings with true systemic bioavailability.

## 7. Structure-Activity Relationships

The complex structural characteristics of macromolecular polysaccharides—including molecular weight, monosaccharide composition, glycosidic linkage patterns, degree of branching, and spatial conformation—strongly influence their specific modes of interaction with host cell surface receptors. This foundational structure–activity relationship paradigm is widely recognized in natural polymer pharmacology [15]. For SFP, key structural parameters—including molecular weight, monosaccharide composition, glycosidic linkage patterns, and higher-order conformation—appear to contribute significantly to their pharmacological efficacy [23]. Detailed structural elucidations of specific SFP fractions isolated by sequential chromatography reveal a clear functional divergence between neutral and acidic components. Neutral polysaccharides, such as SFNP-1 and SFNP-2, which are primarily characterized by (1→4)-linked glucose backbones with sparse branching, exhibit pronounced immunostimulatory potential by significantly enhancing the proliferation of RAW 264.7 macrophages [71]. However, these neutral glucans show a notable lack of direct anti-inflammatory activity, suggesting that their primary role is systemic immune modulation rather than the suppression of pro-inflammatory mediators. Conversely, acidic fractions identified as pectin-type polysaccharides (e.g., SFAP-1 and SFAP-2) consist of *α*-(1→4)-linked galacturonic acid chains with high degrees of methylation (reaching 57.4% and 55.2%, respectively). Despite their pectin-like structure, these acidic components fail to inhibit lipopolysaccharide (LPS)-induced nitric oxide (NO) release and may even stimulate inflammatory markers at higher concentrations [18,83]. This functional profile indicates that the specific esterification patterns and molecular weight distributions of SFP acidic fractions limit their anti-inflammatory utility, implying that the traditional anti-inflammatory reputation of crude *S. flavescens* extracts may be primarily attributed to co-existing alkaloids rather than these macromolecular sugars [6].

Furthermore, the structure–activity paradigm of SFP can be strategically remodeled through targeted chemical modifications. Sulfation has emerged as a particularly effective approach to amplify the bioactivity of native SFP. The introduction of negatively charged sulfate groups—exemplified by derivatives like SFHS (degree of substitution is 1.22)—fundamentally alters the steric conformation and polyanionic charge density of the carbohydrate chain. Based on current experimental observations, these modifications are associated with an increased capacity to scavenge free radicals, inhibit the proliferation of HepG2 tumor cells, and modulate immune responses. While enhanced binding affinity to immune receptors such as TLR4 is frequently proposed in the literature, it is important to note that direct empirical proof of precise binding remains limited [74]. This enhanced interaction is widely hypothesized to stem from electrostatic interactions and structural mimicry of endogenous glycosaminoglycans, which serve as a plausible theoretical model for receptor-mediated signaling rather than a fully verified molecular mechanism.

Polysaccharide modification theory posits that the introduction of strongly electronegative sulfate groups alters chain flexibility and polyanionic charge density, leading to the hypothesis that such molecules may mimic endogenous glycosaminoglycans [58,84]. Within this extrapolated framework, sulfated SFP (e.g., the SFHS derivative with a substitution degree of 1.22) exhibit significantly enhanced antioxidant, targeted antitumor, and macrophage-activating activities, which are mechanistically modeled on enhanced spatial electrostatic affinity for receptors like TLR4. However, these structure–activity correlations—particularly regarding the exact molecular choreography of TLR4 oligomerization and glycosaminoglycan-like behavior—remain largely theoretical extrapolations derived from general polysaccharide pharmacology rather than direct, site-specific experimental confirmation in SFP [85]. Consequently, while these models provide valuable guiding hypotheses for future investigation, rigorous site-directed biophysical and structural studies are ultimately required to validate these proposed receptor recognition pathways.

While sulfation is well-characterized, empirical studies on other modifications of SFP (e.g., acetylation, phosphorylation, and carboxymethylation) remain unreported. However, extrapolating from other botanical polysaccharides, such modifications could critically alter the macromolecules’ solubility, negative charge density, and chain lipophilicity. Consequently, interaction with immune receptors like TLR4 likely extends beyond simple electrostatic affinity; these specific modifications may create precise steric fits and novel hydrogen-bonding networks within the TLR4/MD-2 complex, serving as specific physical triggers for receptor oligomerization.

## 8. Toxicology

A critical aspect of advancing any natural product toward clinical application is the rigorous evaluation of its toxicological profile. While the crude extracts and alkaloid fractions of *S. flavescens*, particularly matrine and oxymatrine are heavily restricted in clinical settings due to their dose-dependent hepatic, renal, and neurological toxicities, the macromolecular polysaccharides extracted from the same plant present a starkly contrasting and highly favorable safety paradigm [86,87]. SFP generally exhibit low intrinsic toxicity and a high safety profile under test conditions. Their biological activities, such as antitumor and anti-inflammatory effects, are typically not achieved through direct cytotoxicity but are closely linked to the host immunomodulatory functions. An early study on the SFP fraction SFPW1 found that, in an in vitro cell model, this component did not exhibit direct cytotoxicity against H22 tumor cells, implying that it does not directly kill tumor cells. However, in subsequent in vivo experiments, SFPW1 effectively inhibited tumor growth and prolonged survival in tumor-bearing mice. These effects were primarily attributed to the enhancement of immune functions, including the promotion of splenocyte proliferation and the activation of macrophages [81].

In terms of in vivo systemic toxicity, the current literature frequently reports high LD50 and No-Observed-Adverse-Effect Level (NOAEL) values for *S. flavescens* botanical preparations. However, it must be emphasized that these toxicological parameters are exclusively derived from whole-root extracts or mixed carbohydrate-rich fractions [88]. These conclusions cannot, and should not, be generalized to purified SFP. To date, there are absolutely no reported in vivo toxicological studies specifically evaluating highly purified, chemically characterized SFP preparations. While preliminary in vitro assessments of isolated SFP fractions indicate favorable cytocompatibility, definitive in vivo safety data for purified SFP is currently entirely absent from the literature. Therefore, conducting systematic in vivo toxicological evaluations on isolated and structurally defined SFP fractions represents an urgent and mandatory prerequisite before any future clinical translation.

Initial toxicological evaluations indicate that SFP generally present a milder safety profile than their alkaloid counterparts. Nevertheless, declaring a definitive “high safety profile” for SFP remains premature given the limited availability of comprehensive in vivo toxicity data. A critical factor often overlooked in these safety assessments is the rigorous quality control of the raw material. *S. flavescens* is renowned for its abundant quinolizidine alkaloids (e.g., matrine and oxymatrine) and prenylated flavonoids. During SFP extraction, trace amounts of these highly bioactive secondary metabolites can easily be co-extracted as impurities. If not rigorously removed through advanced purification techniques—such as repeated deproteinization, dialysis, and ion-exchange chromatography—these non-carbohydrate impurities could drastically confound and alter the intrinsic toxicological and pharmacological profiles of the extracts. Therefore, establishing standardized quality control protocols to continuously monitor and eliminate alkaloid and flavonoid residues is an absolute prerequisite for future SFP research.

## 9. Potential Applications and Translational Prospects

Despite the wide array of pharmacological efficacies and promising therapeutic potential presented in this review, the prospect of SFP in practical clinical applications must be approached with objective caution. It is critical to emphasize that the current body of evidence supporting the efficacy of SFP is almost exclusively derived from preclinical stages, predominantly relying on in vitro cell models and in vivo murine models. Extrapolating these preclinical findings directly to human therapeutic outcomes remains speculative. The complex human gastrointestinal environment, diverse gut microbiota, and complicated pharmacokinetic barriers pose significant challenges to the clinical translation of these macromolecules. Moving forward, the field must transition from basic pre-clinical explorations to rigorously designed, randomized, double-blind human clinical trials to definitively establish the true therapeutic efficacy, optimal dosing regimens, and long-term clinical safety of SFP.

In preclinical models of integrative oncology, SFP show promise as immunomodulatory adjuvants. In vitro and in vivo studies indicate that by regulating cytokine balances and activating macrophages, they may help reconstruct the immunosuppressed tumor microenvironment, suggesting a potential to synergize with conventional chemotherapy to alleviate leukopenia [89]. Beyond oncology, these polysaccharides exhibit hepatoprotective properties relevant to managing chronic inflammatory conditions. By inhibiting inflammatory signaling cascades and boosting endogenous antioxidant defenses, they mitigate drug- and viral-induced hepatic damage, making them promise therapeutic candidates for chronic hepatitis and metabolic syndromes [34]. Ultimately, their multi-target and low-toxicity profiles establish them as sophisticated biological response modifiers. The continued structural elucidation and clinical validation of these natural macromolecules provide a robust foundation for developing next-generation, botanical-based immunomodulatory therapeutics [90].

In addition to the aforementioned clinical application potential, based on their antioxidant and immunomodulatory properties, SFP also demonstrate potential applications in functional foods, natural preservatives, and cosmetic additives [91]. For example, they can serve as natural antioxidants to delay food oxidation and spoilage or be added to health supplements as immunomodulatory components [92]. Furthermore, in the agricultural sector, plant-derived polysaccharides are commonly used as plant immunostimulants; SFP may also play a role in green agriculture [93]. However, research in this area remains limited and warrants further exploration. Overall, SFP hold broad application prospects in the pharmaceutical and health industries; however, current research is largely in the preclinical stage, and future studies should focus on elucidating their mechanisms of action, structure–activity relationships, and clinical translation [92].

## 10. Conclusions and Prospects

As an important bioactive component of *S. flavescens*, SFP have gradually attracted widespread attention in recent years. Existing studies indicate that SFP not only possess various pharmacological activities, including immunomodulatory, antitumor, antioxidant, anti-inflammatory, and lipid-lowering effects, but also that significant progress has been made in their extraction, purification, and structural characterization. However, despite the increasing number of studies each year, research on SFP remains in its preliminary stages, with many unresolved issues.

Collectively, SFP are structurally heterogeneous macromolecules exhibiting pleiotropic bioactivities, particularly in immunomodulation, antitumor effects, and redox regulation, reflecting their multi-target and system-level modes of action. However, current research remains constrained by several critical limitations. The lack of standardized extraction and purification protocols leads to pronounced sample heterogeneity, thereby compromising reproducibility and cross-study comparability. Moreover, structural characterization remains insufficient at the level of fine architecture and higher-order conformation, which restricts a comprehensive understanding of structure–activity relationships. Notably, their bioactivities appear to be highly context-dependent, governed by factors such as molecular weight distribution, monosaccharide composition, branching patterns, and chemical modifications, further complicating mechanistic interpretation. In addition, most studies are limited to in vitro and animal models, with a paucity of systematic pharmacokinetic data and clinical validation, thus hindering their translational potential.

Future investigations should prioritize the integration of advanced multi-scale structural elucidation techniques with mechanism-driven bioactivity evaluation to establish a clearer structure–function paradigm. Controlled modification strategies, such as sulfation and acetylation, may facilitate the identification of key functional motifs and enhance bioactivity. Increasing attention should also be directed toward pharmacokinetic profiling, in vivo target identification, and the exploration of host–microbiota interactions to better understand their systemic effects. Importantly, the development of standardized preparation procedures and robust quality control systems will be essential to ensure consistency and scalability. Finally, well-designed clinical studies are required to substantiate their efficacy and safety, ultimately promoting the development of SFP as promising bioactive agents in pharmaceutical and nutraceutical applications.

## Figures and Tables

**Figure 1:**
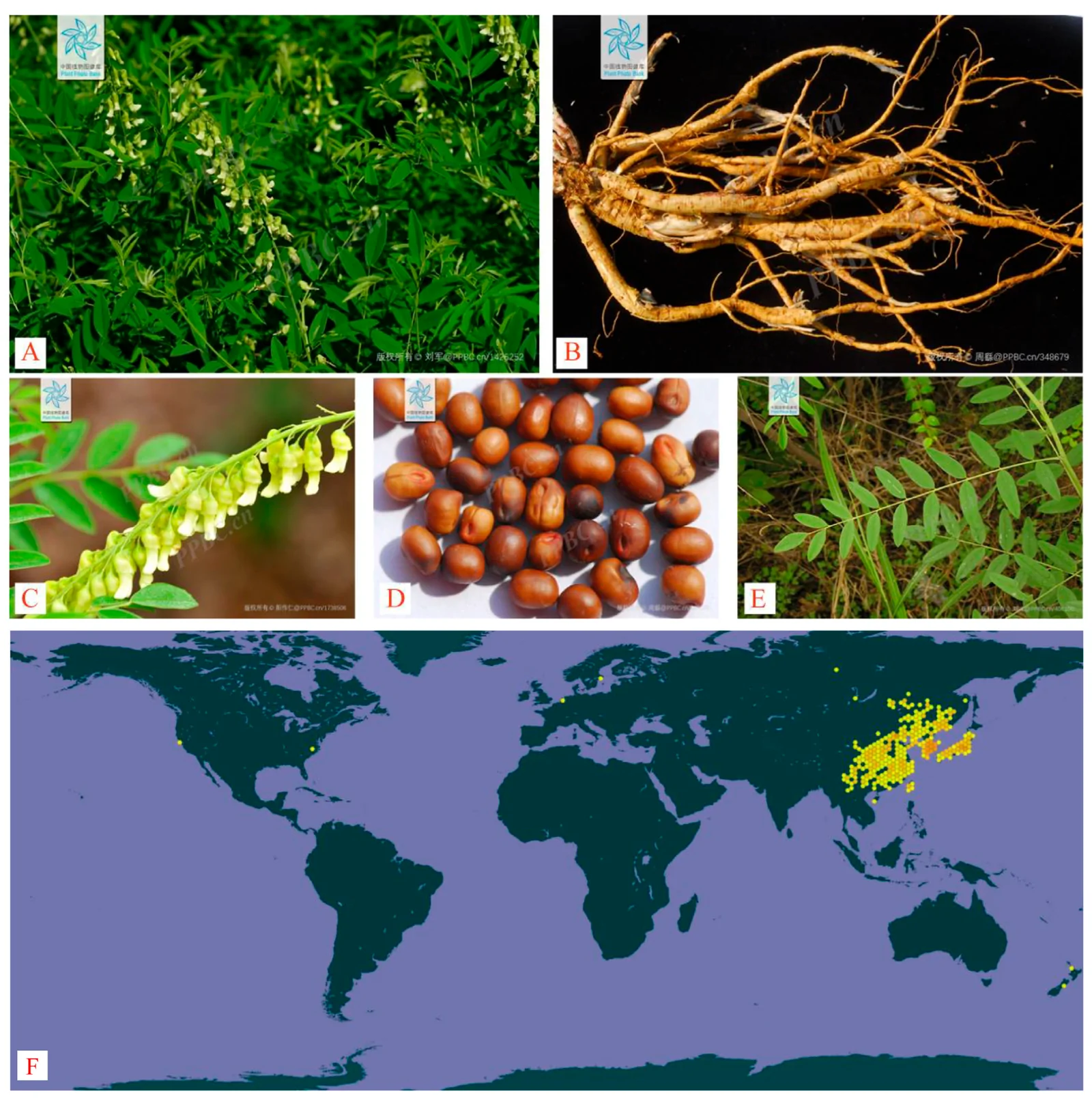
Images of *S. flavescens* (Reprinted/adapted with permission from the Plant Photo Bank of China (PPBC). Copyright [2009], [Yao Zhou].). (**A**) The aerial parts of *S. flavescens*. (**B**) The root of *S. flavescens*. (**C**) The flowers of *S. flavescens*. (**D**) The seeds of *S. flavescens*. (**E**) The leaves of *S. flavescens*. (**F**) Images of Sophora flavescens were accessed through the Global Biodiversity Information Facility (GBIF) network (https://doi.org/10.15468/dl.ydxaem, accessed on 25 September 2026).

**Figure 2:**
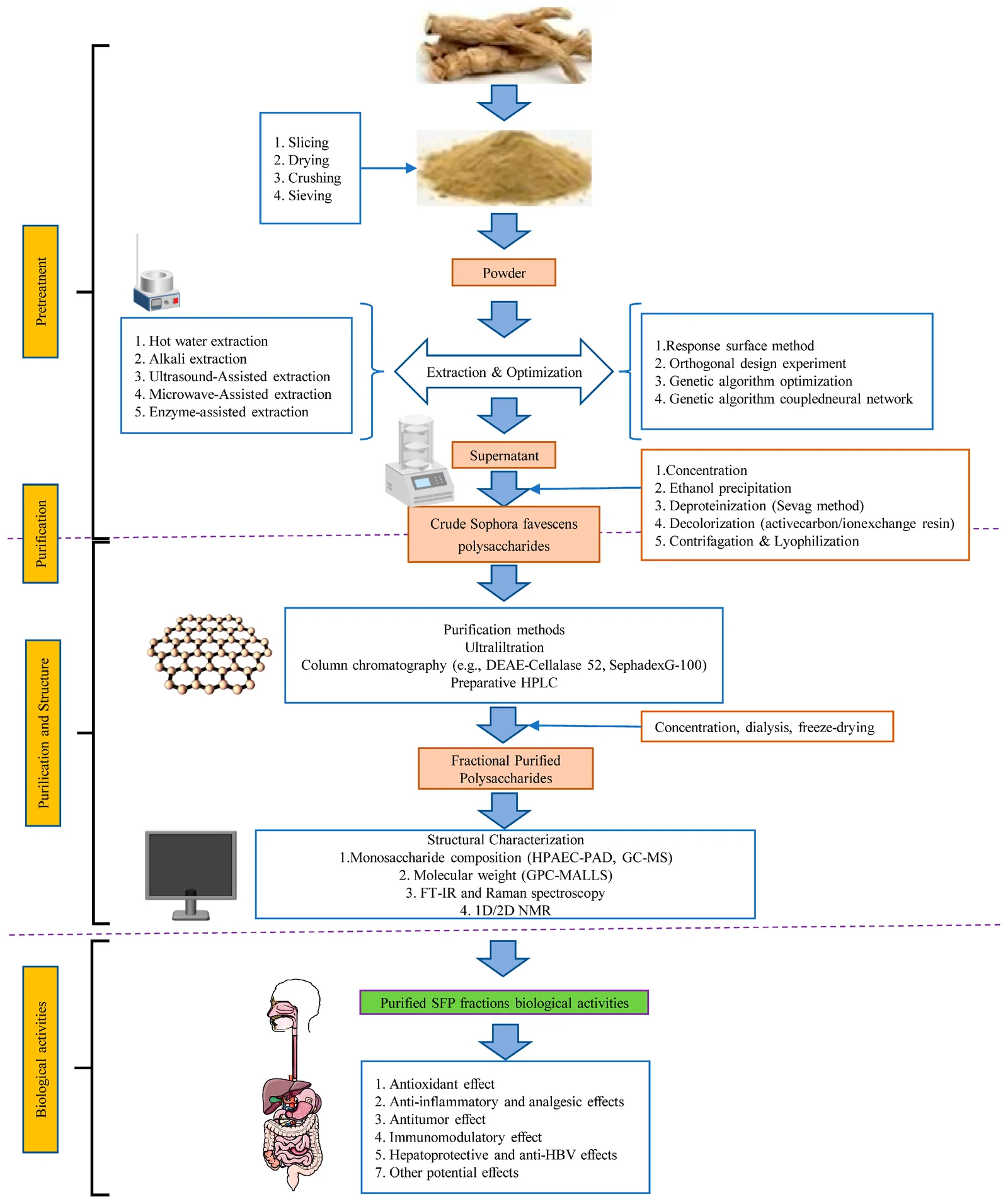
The extraction, purification, structural characteristics, and biological activities of SFP.

**Figure 3:**
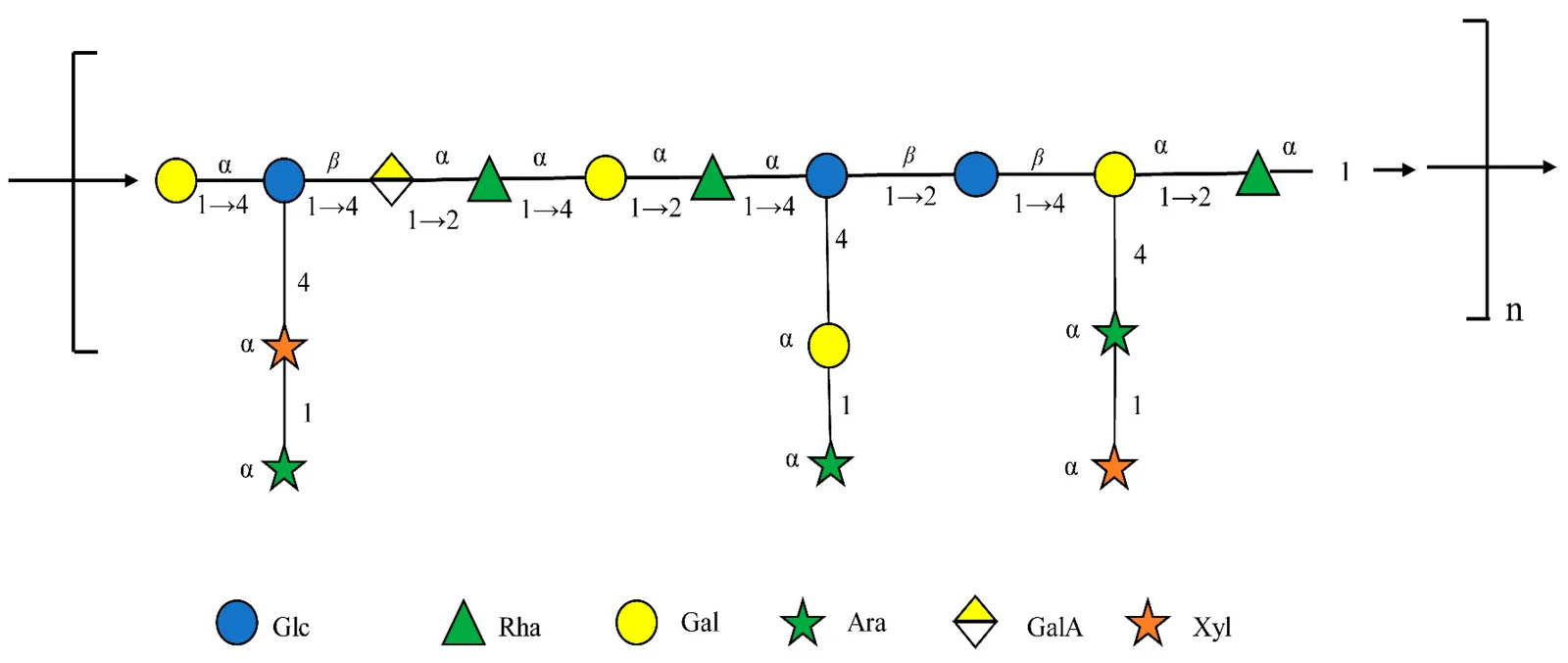
Structure of well-studied SFP.

**Figure 4:**
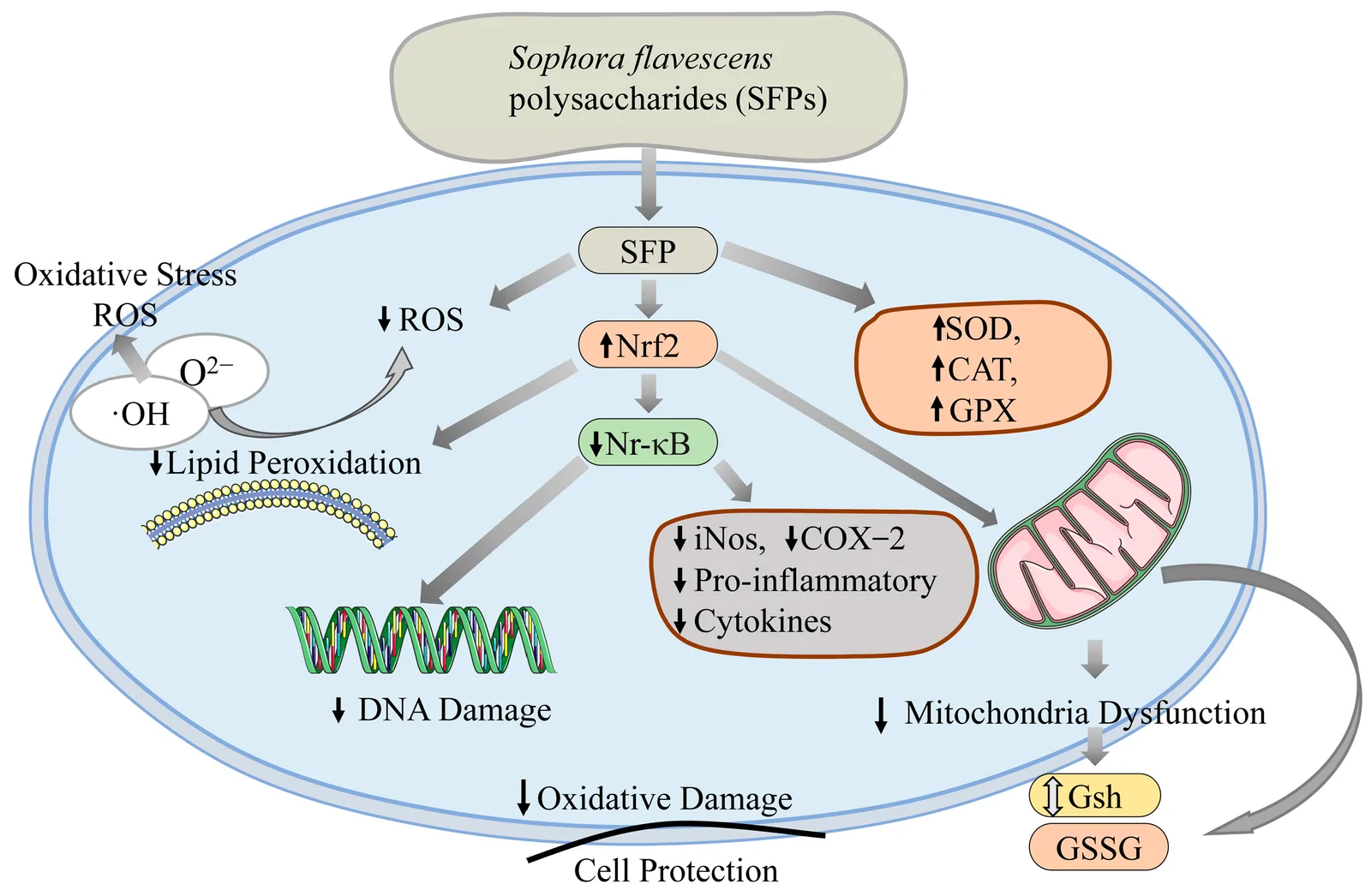
The antioxidant mechanism of *S. flavescens* polysaccharides.

**Figure 5:**
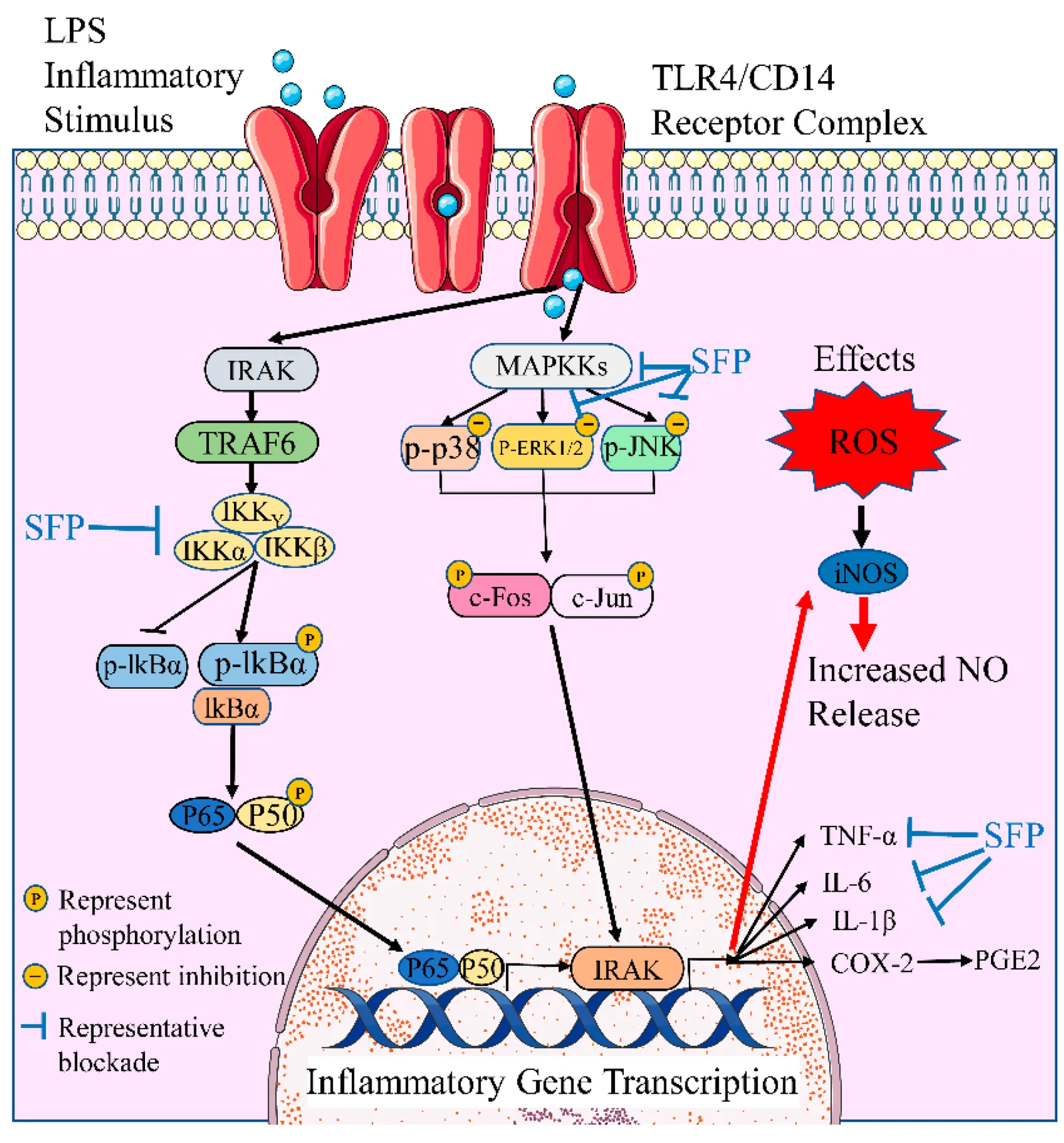
The Anti-Inflammatory Mechanism of SFP.

**Figure 6:**
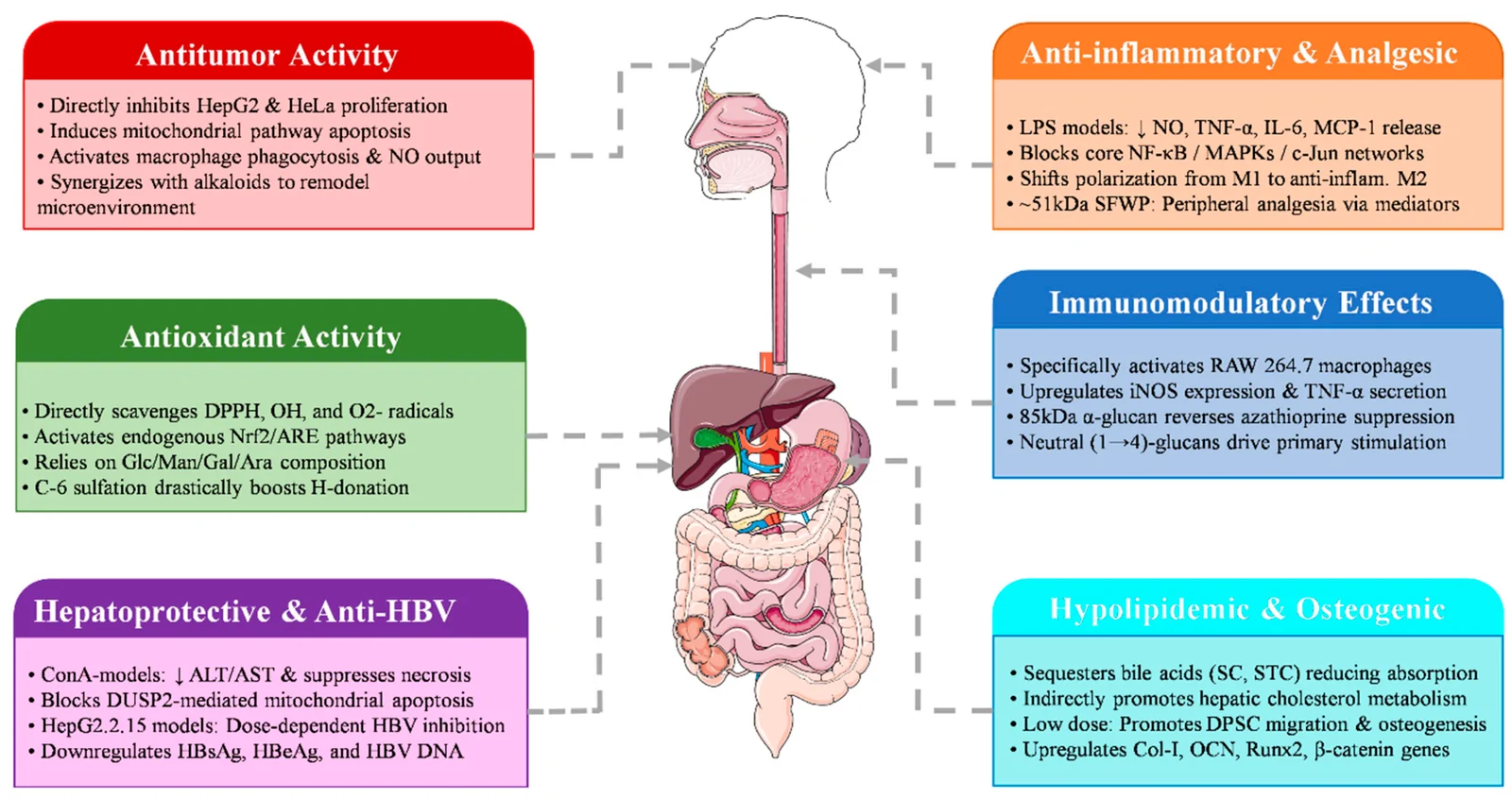
Main pharmacological activities of SFP.

**Table 1:** Extraction and purification of SFP.

Extraction Method	Optimal Parameters	Purification Process	Yield (%)	Cost-Effectiveness Synthesis	Ref.
Hot water extraction	85 °C, 3.5 h, solid–liquid ratio 1:35 g/mL, 3 times	Ethanol precipitation (1:4, *v*/*v*); dialysis (7 kDa); lyophilization	3.07 ± 0.07	High energy and time input with prolonged thermal stress	[19]
Mechanochemistry-assisted extraction	400 rpm, 10 min, powder-to-ball ratio 1:20, loading of 7% Na_2_CO_3_	Ethanol precipitation; Sevag deproteinization; DEAE-Sepharose FF & DEAE-cellulose; Prep-CLC	7.28	Highest efficiency; avoids thermal degradation; minimal time (10 min) and energy cost	[6]
Hot water extraction	100 °C, 1 h each time, twice	Ethanol precipitation; dialysis (≥1) kDa MWC; DEAE-cellulose; Sephadex G-100 chromatography	4.83	Suboptimal yield despite intensive heating	[20]
Hot water extraction	Water extraction and alcohol precipitation	N/A	2.50	Traditional baseline, low recovery	[21]
Water extraction and alcohol precipitation	N/A	Sevag deproteinization; DEAE-52 cellulose column chromatography	N/A	Extended processing time	[22]
Hot water extraction	N/A	Systematic water-soluble fraction isolation	N/A	High thermal energy consumption	[23]

N/A: Not mentioned.

**Table 2:** Pharmacological activities and potential mechanism of SFP.

Activities	Polysaccharides & Structural Features	Models	Dose/Concentration	Potential mechanisms	Ref.
Anti-inflammatory	SFAP-1, SFAP-2: Pectin-type, HG backbone, ~55–57% methylated	In vitro: LPS-induced RAW 264.7 cells	N/A	Exhibits stimulatory effects on inflammatory markers at higher concentrations.	[23]
Osteogenic differentiation	Crude SFP	In vitro: Rat dental pulp stem cells (DPSCs)	N/A	Promotes cell migration and osteogenic differentiation; Up-regulates the expression of Col-I, OCN, Runx2, and *β*-catenin.	[81]
Immunomodulatory	SFP-100 (A/B/C): >100 kDa peak MW SP-1-1/2: ~85 kDa α-glucan core	In vitro/In vivo: Mice spleen lymphocytes & macrophages	50–250 mg/L	Promotes spleen lymphocyte proliferation; Enhances the secretion of cytokines such as IFN-*γ* and TNF-*α*.	[20,59]
Antioxidant	SFHS, SHCS: Sulfated derivatives (e.g., SFHS DS = 1.22)	In vitro: DPPH, ABTS, and OH· free radical models	5 mg/mL	Exhibits strong free radical scavenging capacity; Reduces oxidative stress levels.	[6,19,59]
Antitumor	SHCS, SFHS, SFAS: Sulfated polysaccharides	In vitro: Human hepatoma HepG2 cells	N/A	Inhibits tumor cell proliferation (activity is significantly enhanced by sulfated modification).	[59]
Hypolipidemic	Crude SFP	In vitro: Lipid binding models	10 mg/mL	Demonstrates significant binding capacity to cholesterol and bile salts.	[19]
Hepatoprotective and Anti-HBV	Crude SFP	In vivo: ConA-induced acute liver injury in mice	N/A	Ameliorates liver tissue damage markers; Exhibits significant inhibitory effects against Hepatitis B virus (HBV).	[74]
Antinociceptive	SFWP: ~51 kDa purified fraction	In vivo: Mice models (acetic acid, tail flick, formalin tests)	100–400 mg/kg	Attenuates nociceptive responses; Exhibits both central and peripheral analgesic effects.	[82]

N/A: Not mentioned.

## Data Availability

No data was used for the research described in the article.

## Abbreviations

ALT	Alanine aminotransferase
AST	Aspartate aminotransferase
Ara	Arabinose
ConA	Concanavalin A
DPPH	2,2-diphenyl-1-picrylhydrazyl
DS	Degree of Substitution
EAE	Enzyme-assisted extraction
Gal	Galactose
GalA	Galacturonic acid
Glc	Glucose
HBV	Hepatitis B Virus
HG	Homogalacturonan
HPGPC	High-performance gel permeation chromatography
IL-6	Interleukin-6
LPS	Lipopolysaccharide
MAE	Microwave-assisted extraction
MALLS	Multi-angle laser light scattering
NO	Nitric oxide
NOAEL	No-Observed-Adverse-Effect
RG-I	Rhamnogalacturonan I
Rha	Rhamnose
ROS	Reactive oxygen species
SFAP	*Sophora flavescens* acidic polysaccharides
SFNP	*Sophora flavescens* neutral polysaccharides
SFP	*Sophora flavescens* polysaccharides
TCM	traditional Chinese medicine
TLR4	Toll-like receptor 4
TNF-*α*	Tumor necrosis factor-alpha
